# Clinical Utility of SCALE-B to Predict Hepatitis B Virus Relapse, Hepatitis B Surface Antigen Loss After Antiviral Cessation in Asian Patients After 2-Year Follow-up

**DOI:** 10.3389/fmed.2022.859430

**Published:** 2022-03-24

**Authors:** Apichat Kaewdech, Suraphon Assawasuwannakit, Pimsiri Sripongpun, Naichaya Chamroonkul, Pisit Tangkijvanich, Teerha Piratvisuth

**Affiliations:** ^1^Gastroenterology and Hepatology Unit, Division of Internal Medicine, Faculty of Medicine, Prince of Songkla University, Hat Yai, Thailand; ^2^Department of Medicine, Panyananthaphikkhu Chonprathan Medical Center, Srinakharinwirot University, Nonthaburi, Thailand; ^3^Department of Biochemistry, Faculty of Medicine, Chulalongkorn University, Bangkok, Thailand; ^4^Center of Excellence in Hepatitis and Liver Cancer, Faculty of Medicine, Chulalongkorn University, Bangkok, Thailand; ^5^NKC Institute of Gastroenterology and Hepatology, Songklanagarind Hospital, Prince of Songkla University, Hat Yai, Thailand

**Keywords:** clinical relapse (CR), hepatitis B core-related antigen (HBcrAg), hepatitis B surface antigen (HBsAg), hepatitis B virus ribonucleic acid (HBV RNA), nucleos(t)ide analogues (NAs), cessation, SCALE-B, S-loss

## Abstract

**Background:**

Discontinuation of antiviral therapy in chronic hepatitis B (CHB) patients leads to a higher hepatitis B surface antigen (HBsAg) loss; yet, clinical relapse (CR) may occur. SCALE-B score was developed to predict off-treatment CR; however, validation of SCALE-B beyond a 48-week follow-up is rare. We studied whether SCALE-B and hepatitis B virus ribonucleic acid (HBV RNA) could predict outcomes in CHB patients after a 2-year follow-up.

**Methods:**

A total of 92 Thai CHB patients who stopped antiviral treatment were followed up; baseline characteristics, quantitative hepatitis B surface antigen (qHBsAg), hepatitis B core-related antigen (HBcrAg), and HBV RNA were collected at the time of discontinuation, and SCALE-B scores were calculated. Patients were followed up every 12 weeks for 48 weeks, and then, the intervals were upon primary doctors. Follow-up data regarding virological relapse (VR), CR, and HBsAg loss were obtained.

**Results:**

The median follow-up duration was 142 weeks; the cumulative incidences of VR, CR, and HBsAg loss were 65.2, 33.7, and 7.6%, respectively. After 48 weeks, VR and CR plateaued, but HBsAg loss increased from 2.2 to 7.6%. According to the SCALE-B strata, VR, CR, and HBsAg loss were significantly different. The highest stratum (≥ 320) was associated with higher VR, CR, and lesser HBsAg loss when compared to the lowest stratum, with adjusted hazard ratios of 5.0 (95% CIs: 1.8–14.4), 10.44 (95% CIs: 1.4–79.1), and 0.04 (95% CIs: 0.004–0.43), respectively.

**Conclusion:**

At a median follow-up of 2.5 years after discontinuing therapy, HBsAg loss in Thai patients was found to increase over time. SCALE-B is a valuable tool for predicting CR, VR, and HBsAg loss; HBV RNA is not significantly associated with long-term outcomes.

**Clinical Trial Registration:**

[www.ClinicalTrials.gov], identifier [TCTR20180316007].

## Introduction

Apart of perpetually suppressible viral load, the ultimate goal of hepatitis B treatment is to achieve hepatitis B surface antigen (HBsAg) loss with or without an appearance of hepatitis B surface antibody, which is a so-called functional cure (1, 2). However, this endpoint is uncommonly achieved by long-term antiviral treatment, and there were mathematical models published earlier that HBsAg clearance was predicted to attain at 36–52 years after nucleos(t)ide analogs (NAs) therapy (3, 4), as NAs cannot inhibit viral transcription or eliminate closed-covalent circular DNA (cccDNA), but only inhibit the reverse transcriptase activity *via* viral polymerase (2).

Several international hepatology societies suggest that NAs treatment can be stopped in selected patients, particularly those without cirrhosis and with persistent normal alanine aminotransferase (ALT) and undetectable hepatitis B virus (HBV) DNA (5–7). The advantages of NAs discontinuation include increased HBsAg loss, improved adherence with a finite duration, decreased medication side effects in long-term use, and lower cost (8). However, some critical drawbacks may be encountered, for example, severe clinical relapse (CR) which can lead to liver decompensation or death (9–11). Patients that experience relapse might also have a higher risk of developing hepatocellular carcinoma (HCC), although the supporting evidence is limited (12).

Choosing candidates who can stop NAs treatment is challenging as physicians need to balance the risks and benefits; the best candidates are those with low risk of CR and a high chance of achieving HBsAg loss. Recent studies have reported a number of HBV biomarkers for predicting outcomes following treatment discontinuation, which includes quantitative hepatitis B surface antigen (qHBsAg) (13), hepatitis B core-related antigen (HBcrAg) (9, 10, 14–16), and serum hepatitis B virus ribonucleic acid (HBV RNA) (17–20), and also predictive models. The SCALE-B score (21) [HB**s**Ag level (S), HB**c**rAg (C), **a**ge (A), A**L**T (L), and t**e**nofovir (E) used for H**B**V (B) treatment] is calculated as follows: HBsAg (log_10_ IU/ml) + 20 × HBcrAg (log_10_ U/ml) + 2 × age (year) + ALT (U/L) + 40 for the use of tenofovir (16). The SCALE-B model was specifically developed to predict CR in Taiwanese patients who stopped NAs. The score was classified into three strata based on the risk of developing CR: high (≥320 points), intermediate (260–320 points), and low (<260 points). Despite a recent study validating the SCALE-B score (10), it remains unclear whether SCALE-B can predict off-therapy outcomes beyond a 1-year follow-up.

In this study, we aimed to evaluate the clinical utility of SCALE-B to identify the candidate patients who can stop NAs to maximize the benefits of HBsAg loss and minimize CR in patients with a longer follow-up duration.

## Materials and Methods

### Patients and Study Design

This is a retrospective study of long-term follow-up outcomes of patients participating in the HBV biomarkers stop study; the detailed methods and results of the primary study have been published (9). In brief, non-cirrhotic patients with chronic hepatitis B (CHB) treated with NAs who met the criteria for treatment discontinuation according to the Asian Pacific Association for the Study of the Liver (APASL) (5) guidelines were invited and informed about the study, and patients who agreed to stop NAs and participate in the study were followed up for clinical and laboratory tests every 12 weeks for 48 weeks in the primary study. After 48 weeks following NAs cessation, the patients were followed up every 12–24 weeks, with the frequency of laboratory tests determined by their primary physicians. Patients with advanced fibrosis (≥F3 fibrosis by METAVIR or liver stiffness ≥11 kPa by transient elastography) either at baseline before NAs initiation or at the time of stopped NAs were excluded.

At the time of stopping NAs, liver biochemistry parameters and also levels of HBV DNA, qHBsAg, HBcrAg, and HBV RNA were measured. Following NAs discontinuation, the liver biochemistries and HBV DNA were measured every 12 weeks for 48 weeks, whereas qHBsAg level was measured in every patient at 48 weeks (the end of follow-up of the primary study) (2). After 48 weeks off-therapy, the patients were followed up by their primary physicians; the timing of follow-up varied, but the clinical and laboratory tests were usually conducted every 12–24 weeks.

Eligible patients were recruited between February 2018 and August 2019. Patients with active malignancy, which includes HCC, or concurrent treatment with any immunosuppressive therapy were excluded. In this study, we collected the follow-up data from patients until 31 August 2021.

Based on the data at NAs discontinuation [end of the NAs treatment (EOT)], we calculated SCALE-B score using the formula stated earlier. The outcomes of interest were virological relapse (VR), which was defined by serum HBV DNA levels higher than 2,000 IU/ml; CR, which was defined as a VR plus ALT level that was more than two times the upper limit of normal (ULN; 33 U/L for men and women) between each SCALE-B stratum; and HBsAg clearance, which was defined by undetectable levels of qHBsAg (<0.05 IU/ml). Retreatment was initiated in patients developed CR plus one of the following criteria: total bilirubin > 1.5 mg/dL, prolong prothrombin time >2 s, serum ALT >10 times of ULN or >2 times but ≤10 times for ≥4 weeks as prespecified in previous study (9).

All patients provided informed consent before participating in the primary study. In this study, we acquired data solely retrospectively; therefore, the informed consent requirement was waived. The protocol for this study was approved by the Faculty of Medicine’s Institutional Review Board and Ethics Committee, Prince of Songkla University, Thailand (REC: 64-379-14-1). The study was conducted under the ethical guidelines of the 1975 Declaration of Helsinki.

### Laboratory Assays

The i2000 Chemical Luminescent Immuno-analyzer (Abbott, Chicago, IL, United States) was used to quantify HBsAg; the lower limit of detection (LLoD) was 0.05 IU/ml. The COBAS TaqMan assay (Roche Diagnostics, Branchburg, NJ, United States) was used to quantify HBV DNA with the LLoD of 10 IU/ml. The Lumipulse G HBcrAg assay (Lumipulse System; Fujirebio, Tokyo, Japan) was used to quantify serum HBcrAg levels with the LLoD of 3 log10 U/ml. A droplet digital PCR (Bio-Rad, Hercules, CA, United States) was used to assess serum HBV RNA levels; the LLoD was 2.0 log10 copies/ml, as previously described (22).

### Statistical Analyses

To calculate sample size, we hypothesized that the CR would occur in 35% of the patients after 2 years of follow-up with the precision of 0.1 and SCALE-B score would have a sensitivity of 92%. At the alpha level of 0.05, a sample size of at least 81 patients was requisite, in which 92 patients of the original HBV biomarkers stop study will cover the required sample size.

For the statistical analyses of the outcomes of interest, continuous variables such as age, ALT, and HBV biomarkers that include qHBsAg, HBcrAg, and HBV RNA were expressed as mean (±SD) or median [interquartile range (IQR)], as appropriate. Categorical variables were expressed as number and percentages. According to SCALE-B strata, the Chi-square test or, when appropriate, Fisher’s exact test was used to compare categorical variables and an ANOVA or Kruskal–Wallis test for comparing continuous variables. The survival probability of being free of VR and CR and the cumulative incidence of HBsAg loss were calculated by Kaplan–Meier method and compared with the log-rank test. A Cox regression analysis was used to estimate the hazard ratios and 95% confidence intervals (CIs) of variables associated with VR, CR, and HBsAg loss and adjusted for other potential factors. All statistical analyses were performed with R software, version 4.1.0 (R Foundation, Austria). A two-sided *p*-value of <0.05 was considered statistically significant.

## Results

### Baseline Clinical Characteristics

We stratified patients into three SCALE-B score strata as follows: <260 points (low SCALE-B group; *n* = 14), between 260 and 320 points (intermediate SCALE-B group; *n* = 41), and ≥320 points (high SCALE-B group; *n* = 37); the baseline characteristics of patients in each stratum are shown in Table 1. As expected, the patients with a higher SCALE-B score had higher levels of ALT, qHBsAg, HBcrAg, and tenofovir disoproxil fumarate (TDF) use at the end of treatment because these variables were the parameters used in the SCALE-B formula; however, the mean age, sex, proportion of hepatitis B e-antigen (HBeAg)-negative patients, and HBV RNA levels were not significantly different across the groups. Although all patients had undetectable HBV DNA, the EOT HBV RNA was still detected in all SCALE-B score groups.

**TABLE 1:** Baseline clinical characteristics of the cohort stratified by SCALE-B scores (*n* = 92).

Characteristic	SCALE-B < 260 (*N* = 14)	SCALE-B 260-320 (*N* = 41)	SCALE-B ≥ 320 (*N* = 37)	*p*-Value
Age, years, mean (SD)	53.5 (8.3)	55.0 (9.3)	56.1 (10.4)	0.666
Male sex, no. (%)	8.0 (57.1)	26.0 (63.4)	25.0 (67.6)	0.780
HBeAg-negative, no. (%)	11.0 (78.6)	32.0 (78.0)	29.0 (78.4)	0.999
**End of treatment levels**				
Alanine aminotransferase (ALT), U/L	17.0 (10.8–18.0)	19.0 (16.0–23.0)	25.0 (20.0–35.0)	<0.001
qHBsAg, IU/ml	75.4 (12.3–224.3)	702.0 (280.4–2172.8)	2,574.1 (853.8–5,723.9)	<0.001
qHBsAg ≥ 100 IU/ml, no. (%)	5.0 (35.7)	35.0 (85.4)	34.0 (91.9)	<0.001
HBcrAg, log_10_ U/ml	2.7 (2.1–3.6)	2.9 (2.7–3.2)	3.8 (3.3–4.5)	<0.001
HBcrAg ≥ 3 log_10_ U/ml, no. (%)	5.0 (35.7)	20.0 (48.8)	33.0 (89.2)	<0.001
HBV RNA, copies/ml	0 (0–248.5)	136.6 (0–514.5)	213.5 (0–1,708)	0.069
HBV RNA ≥ 2 log_10_ copies/ml, no. (%)	4.0 (28.6)	21.0 (51.2)	22.0 (59.5)	0.144
**Last antiviral drug(s) taken, no. (%)**				0.027
LAM	8.0 (57.1)	21.0 (51.2)	12.0 (32.4)	
LAM + TDF	1.0 (7.1)	5.0 (12.2)	14.0 (37.8)	
ETV	3.0 (21.4)	7.0 (17.1)	3.0 (8.1)	
LdT	1.0 (7.1)	6.0 (14.6)	2.0 (5.4)	
TDF	1.0 (7.1)	1.0 (2.4)	6.0 (16.2)	
LAM + ADV	0 (0)	1.0 (2.4)	0 (0)	
TDF user, no. (%)	2.0 (14.3)	6.0 (14.6)	20.0 (54.1)	<0.001

*ADV, adefovir; ALT, alanine aminotransferase; ETV, entecavir; HBcrAg, hepatitis B core-related antigen; HBeAg, hepatitis B e-antigen; HBV, hepatitis B virus; LAM, lamivudine; LdT, telbivudine; qHBsAg, quantitative hepatitis B surface antigen; TDF, tenofovir disoproxil fumarate. Data are expressed as medians with interquartile ranges except where noted.*

### Rates of Hepatitis B Surface Antigen Loss and Hepatitis B Virus Relapse After Nucleos(t)ide Analogues Discontinuation

**FIGURE 1:**
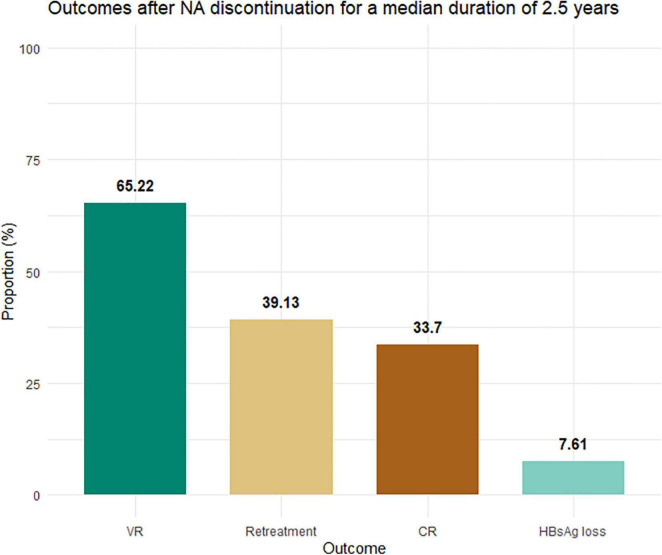
Proportion of patients experiencing virological relapse, CR, retreatment, and HBsAg loss. NAs, nucleos(t)ide analogs; ULN, upper limit of normal (33 U/L for men and women). Virological relapse was defined by serum HBV DNA levels >2,000 IU/ml. CR was defined as virological relapse plus serum alanine aminotransferase levels >2× ULN. HBsAg loss was defined by an undetectable HBsAg level.

The median follow-up duration for all patients in the cohort was 142 (IQR: 126–158) weeks. Interestingly, the rate of HBsAg loss continuously increased over time, from 2 out of 92 patients (2.2%) at week 48 to 7 patients (7.6%) at the end of follow-up (Figure 1). Whereas the new occurrences of VR and CR were rarely observed after 48 weeks following treatment discontinuation, the VR and CR were 63 and 33.7% at 48 weeks and 65.2 and 33.7% at the end of follow-up, respectively. Among those who experienced VR or CR, the NAs treatment was reintroduced in 39.1%.

### Association Between the SCALE-B Score and Hepatitis B Virus Relapse and Hepatitis B Surface Antigen Loss

**FIGURE 2:**
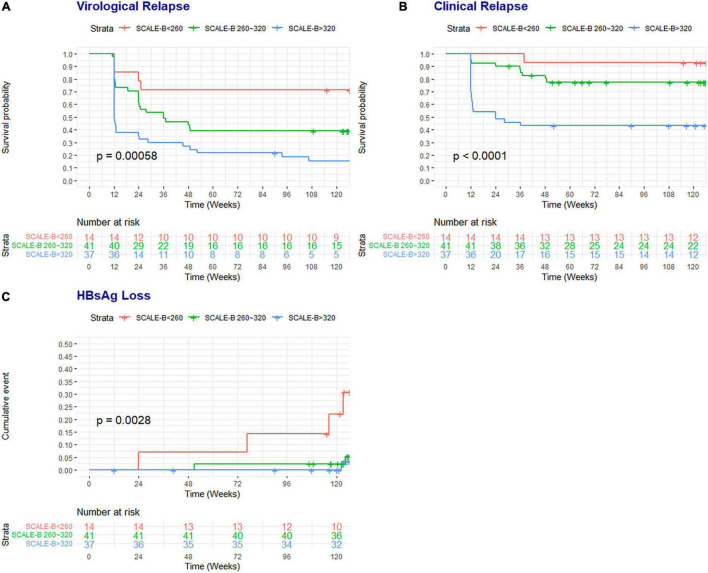
The survival probability of patients free of virological relapse and CR and the cumulative rates of HBsAg loss according to the SCALE-B strata. **(A)** Virological relapse, **(B)** CR, and **(C)** HBsAg loss. SCALE-B scores were calculated as HBsAg (log_10_ IU/ml) + 20 × HBcrAg (log_10_ U/ml) + 2 × age (year) + ALT (U/L) + 40 for the use of tenofovir, where HBsAg level (S), HBcrAg (C), age (A), ALT (L), and tenofovir (E) use for HBV (B) treatment. The SCALE-B score is divided into three groups: low SCALE-B score (<260 points), intermediate SCALE-B score (between 260 and 320 points), and high SCALE-B score (≥320 points).

Figure 2 illustrates the Kaplan–Meier survival curves of clinical outcomes of patients in each SCALE-B stratum. There were significant differences in the rates of VR (*p* < 0.001), CR (*p* < 0.001), and HBsAg loss (*p* < 0.001) among different SCALE-B groups. For example, at 96 weeks, 71.4% (95% CIs: 51.3–99.5%) of patients in the low SCALE-B group remained free of VR, whereas 39.0% (95% CIs: 26.6–57.2%) and only 18.5% (95% CIs: 9.4–36.7%) of patients in the intermediate and high SCALE-B groups were VR free, respectively. The rate of HBsAg loss at 96 weeks was observed in 14.3, 2.4, and 0% of patients in the low, intermediate, and high SCALE-B groups, respectively.

**FIGURE 3:**
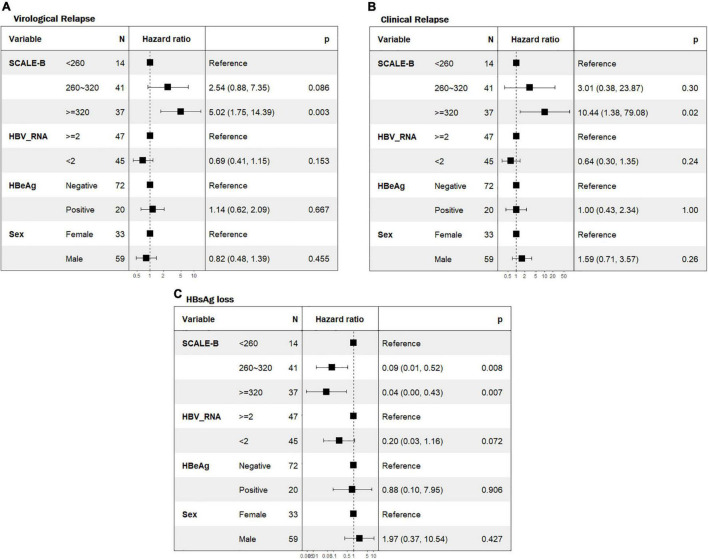
Forest plot of the multivariate Cox regression analyses for predicting virological relapse, CR, and HBsAg loss. **(A)** VR, **(B)** CR, and **(C)** HBsAg loss. SCALE-B scores were calculated as HBsAg (log_10_ IU/ml) + 20 × HBcrAg (log_10_ U/ml) + 2 × age (year) + ALT (U/L) + 40 for the use of tenofovir, where HBsAg level (S), HBcrAg (C), age (A), ALT (L), and tenofovir (E) use for HBV (B) treatment. Adjusted hazard ratios of patients with pretreatment HBeAg status, EOT HBV RNA level, and SCALE-B score. HBeAg, hepatitis B e-antigen; HBV RNA, hepatitis B virus ribonucleic acid.

We then explored the role of SCALE-B strata in the prediction of clinical outcomes following NAs discontinuation using multivariable Cox regression analyses. The results were adjusted for parameters not included in the SCALE-B but potentially linked with the outcomes, which include sex, pretreatment HBeAg status, and EOT HBV RNA. Patients in the high SCALE-B group had a significantly higher risk of VR [adjusted hazard ratio (aHR) 5.02, *p* = 0.003, Figure 3A] and CR (aHR 10.44, *p* = 0.02, Figure 3B) and also a reduced likelihood of achieving HBsAg loss (aHR 0.04, *p* = 0.007, Figure 3C) when compared to those in the low SCALE-B group. However, when adjusted for SCALE-B strata, sex, and HBeAg, HBV RNA was not able to predict clinical outcomes following NAs discontinuation.

When compared between SCALE-B score and HBV RNA individually in predicting CR, the SCALE-B was again significantly better than HBV RNA with the areas under the receiver operating characteristic (AUROC) of 0.81 vs. 0.66 (*p* = 0.044). The ROC curves and diagnostic performance (sensitivity, specificity, positive predictive, and negative predictive values) of SCALE-B and HBV RNA in predicting CR at 96 weeks are shown in Supplementary Figure 1 and Supplementary Table 1.

### Clinical Safety After Nucleos(t)ide Analogues Discontinuation

In the course of follow-up, none of the patients died or developed HCC or liver failure. However, there were two patients developed severe hepatitis flare at week 12 after NAs cessation and required hospitalization. Of note, after retreatment with NAs, clinical jaundice and ALT returned to normal within 12–16 weeks. These two patients were already reported in the primary study (9). After the end of primary study, beyond 48 weeks of follow-up post-NAs discontinuation, there was no additional case of severe hepatitis or liver failure. HBsAg loss was not observed in those patients who required retreatment.

## Discussion

The challenges in NAs discontinuation are high rates of VR, CR, and a difficulty in identifying CHB patients who are likely to achieve functional cure, especially in Asian populations (8), as using only discontinuation criteria recommended by international guidelines does not result in satisfactory off-therapy outcomes (5–7, 23). Recently, the SCALE-B model was developed for Taiwanese patients with CHB, which incorporated variables associated with CR following treatment discontinuation in that cohort (i.e., qHBsAg, HBcrAg, age, ALT at EOT, and TDF use). The score was validated in a small number of Caucasian patients with CHB, which demonstrated that the model could predict only HBsAg clearance but not CR (16, 21). This study is the first to externally validate the clinical utility of SCALE-B after a follow-up period of more than 2 years post-NAs discontinuation, compared with the use of other novel biomarkers such as HBV RNA. Our findings indicated that the SCALE-B score could predict both VR and CR, and also HBsAg loss significantly. When adjusted for the SCALE-B strata, HBV RNA was no longer associated with off-treatment clinical outcomes.

Notably, after off-therapy for over 1 year, VR and CR rates seemed to be plateau; for example, after 2.5 years, the VR rate increased from 63 to 65.22% and the CR rate remained at 33.7% compared with the data at 1 year posttreatment discontinuation in this cohort (9). Nonetheless, our result differed from that of a pooled analysis that reported a gradual increase in VR from 48.6 to 60.7% after 1 and 2 years following treatment discontinuation, respectively (24). However, the definition of VR among studies included in the pooled analysis varied, ranging from 200 to 20,000 IU/ml (24). Of note, careful and frequent monitoring within the first year after stopping NAs may be needed as most cases of HBV relapse occurred within 1 year; less frequent follow-ups may be considered beyond this initial time point.

Beyond 1 year, there was an increase in HBsAg loss from 2.2 to 7.6% in this study (25). This finding is in contrast to that reported in a large Taiwanese cohort, wherein the annual incidence of HBsAg clearance was 1.78% (11); a high incidence of HBsAg seroclearance was reported in a high proportion of Caucasians (26, 27). Hypothetically, the HBV genotype (e.g., genotype C) and the duration of perinatal infectivity in Asians with CHB may play a major role in the difficulty in acquiring functional cure status (28). Interestingly, our results showed that HBsAg loss after 1-year off-treatment in Asian patients was not as trivial as previously reported (11, 29). Thus, the “wait and see strategy” may be used for thorough monitoring and does not eliminate the possibility of functional cure in some patients.

Our study confirmed the clinical utility of SCALE-B for the prediction of VR, CR, and HBsAg loss in Asians with CHB for a median duration of 2.5 years after treatment discontinuation. The low SCALE-B stratum exhibited a lower chance of VR and CR and a higher opportunity of HBsAg clearance impressively. Furthermore, we found that serum HBV RNA at EOT was not effective at predicting VR, CR, and HBsAg loss when used in addition to SCALE-B scoring.

SCALE-B comprises of multiple variables known to have a significant association with clinical outcomes after NAs discontinuation, which might explain its strong predictive ability for VR, CR, and HBsAg loss. For instance, a prior systematic review showed that a low level of qHBsAg, particularly <100 IU/ml, was associated with low rates of VR and CR and high HBsAg loss (13). However, the efficiency of using qHBsAg as the sole predictor of HBsAg clearance was not confirmed in a subgroup of Asian patients (30). Serum HBcrAg, another biomarker encompassed hepatitis B core antigen, HBeAg, and p22 protein, is also a potential marker that reflects the intrahepatic transcription of cccDNA (10, 14, 15, 31). TDF, when used as a final antiviral before treatment discontinuation, was reported to be associated with higher and shorter time to relapse than in entecavir users (32, 33).

Serum HBV RNA is an emerging marker used for predicting the phase of HBV infection in patients with CHB and is useful for identifying patients who could benefit from discontinuation of therapy (31). However, there have been some discrepancies in the value of this marker, which are likely due to the different cut-off levels used, the heterogeneity of measurement techniques, and the various subtypes of RNA (20). Off-therapy HBV relapse was recently shown to be as high as 60% in patients treated with entecavir who had undetectable HBV RNA at the EOT (18), which suggests that HBV RNA is a poor surrogate marker when it is used alone.

In addition, the use of combined biomarkers could improve patient evaluation; a recent systematic review demonstrated that the combination of novel biomarkers performed better than the use of a single biomarker (31). For example, using serum HBcrAg and HBV RNA together had a greater accuracy in predicting off-therapy relapse than using serum HBV RNA alone (9, 19, 31, 34). These results are consistent with our findings that the SCALE-B, which involves a combination of several predictive variables, is a better predictive method for off-therapy relapse than HBV RNA, which provides no additional benefit to the SCALE-B score.

The strength of our study is the use of a long-term follow-up cohort to evaluate the clinical utility of novel HBV biomarkers, which include serum qHBsAg and HBcrAg levels incorporated into the SCALE-B score, and also EOT HBV RNA to predict off-therapy relapse and HBsAg clearance, altogether with other baseline clinical characteristics of the patients. However, there were some limitations. We acknowledge the limited number of sample size in our study, as we calculated it for the outcome in the primary HBV biomarker stop study (9). But with the complete data on laboratory and clinical variables at the time of stopping NAs and the longer period of follow-up up to 3.5 years in this study, we consider that the results of this study provide some significant insights into HBsAg loss and other off-therapy clinical outcomes in Asian CHB patients. Additionally, our cohort included only Thai CHB patients who presumably were infected with HBV genotype C based on the geographic prevalence; therefore, the results may not be generalizable to other HBV genotypes (35). Further studies in patients of diverse ethnicities and other HBV genotypes are needed for the broader application of SCALE-B. Another point of concern is that retreatment was initiated in 39% of the patients in our cohort based on prespecified criteria, some of which could have had the additional benefit of HBsAg loss if retreatment had been withheld for longer. As there was a novel concept of the beneficial flare of immune restoration after discontinuation of NAs in patients who had declined qHBsAg level before or during CR, they may achieve spontaneous HBsAg loss without the need of antiviral reintroduction (36). Finally, the qHBsAg was not systematically tested in our patients after 48 weeks following treatment discontinuation, with the follow-up qHBsAg level tested after 1 year based on their primary doctor’s decision. Nevertheless, this implies that the actual number of patients who achieved HBsAg loss might be higher than what we reported.

## Conclusion

In conclusion, our study highlighted the increasing rate of HBsAg loss after 1 year off-treatment in Asian patients, although it was not as high as that in Western countries. We reported a 7.6% HBsAg loss at the median of 2.5-year follow-up period, which was substantially higher than that observed upon continuing NAs treatment in Asian populations. The results also confirmed the utility of SCALE-B score in the prediction of off-therapy HBsAg loss and CR and demonstrated the additional benefit of predicting VR in those considering NAs cessation; serum HBV RNA was not a significant predictive factor for long-term outcomes.

## Data Availability Statement

The data that supports the findings of this study is available on request from the corresponding author.

## Ethics Statement

The studies involving human participants were reviewed and approved by the Faculty of Medicine’s Institutional Review Board and Ethics Committee, Prince of Songkla University, Songkhla, Thailand (REC: 64-379-14-1). Written informed consent for participation was not required for this study in accordance with the national legislation and the institutional requirements.

## Author Contributions

SA, AK, and PS made a substantial contribution to the study concept and design, analysis and interpretation of data, drafting the manuscript, and critical revision of the manuscript. NC, PT, and TP were the senior authors responsible for interpreting data and critically revising the manuscript. All authors contributed to critical revisions and approved the final manuscript.

## Conflict of Interest

TP has received research grants from Gilead Sciences, Roche Diagnostic, Janssen, Fibrogen, and VIR, speaker honoraria from Bristol-Myers Squibb, Gilead Sciences, Bayer, Abbott, Esai, Mylan, Ferring, and MSD. The remaining authors declare that the research was conducted in the absence of any commercial or financial relationships that could be construed as a potential conflict of interest.

## Publisher’s Note

All claims expressed in this article are solely those of the authors and do not necessarily represent those of their affiliated organizations, or those of the publisher, the editors and the reviewers. Any product that may be evaluated in this article, or claim that may be made by its manufacturer, is not guaranteed or endorsed by the publisher.

## Abbreviations

ADV: adefovir
aHR: adjusted hazard ratio
ALT: alanine aminotransferase
APASL: Asian Pacific Association for the Study of the Liver
cccDNA: closed-covalent circular DNA
CHB: chronic hepatitis B
CIs: confidence intervals
CR: clinical relapse
EOT: end of treatment
ETV: entecavir
HBcrAg: hepatitis B core-related antigen
HBeAg: hepatitis B e-antigen
HBsAg: hepatitis B surface antigen
HBV RNA: hepatitis B virus ribonucleic acid
HBV: hepatitis B virus
HCC: hepatocellular carcinoma
IQR: interquartile range
LAM: lamivudine
LdT: telbivudine
LLoD: lower limit of detection
NAs: nucleos(t)ide analogs
qHBsAg: quantitative hepatitis B surface antigen
TDF: tenofovir disoproxil fumarate
ULN: upper limit of normal
VR: virological relapse.
