# Supplementary figures and images for: Clinical Utility of SCALE-B to Predict Hepatitis B Virus Relapse, Hepatitis B Surface Antigen Loss After Antiviral Cessation in Asian Patients After 2-Year Follow-up
Source: Front Med (Lausanne). 2022 Mar 24;9:859430. doi: 10.3389/fmed.2022.859430 (PMC8987127; doi:10.3389/fmed.2022.859430)

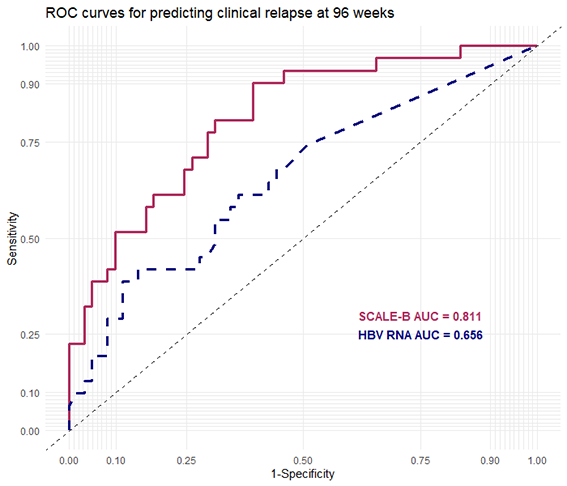

Supplement: Supplementary file 1 [file Image_1.TIFF]
