# Supplementary material for: Clinical Utility of SCALE-B to Predict Hepatitis B Virus Relapse, Hepatitis B Surface Antigen Loss After Antiviral Cessation in Asian Patients After 2-Year Follow-up
Source: Front Med (Lausanne). 2022 Mar 24;9:859430. doi: 10.3389/fmed.2022.859430 (PMC8987127; doi:10.3389/fmed.2022.859430)
Supplement: Supplementary file 2 [file Table_1.DOCX]

**Supplementary Table 1.** Sensitivity, specificity, and predictive values of the SCALE-B and the end of treatment serum HBV RNA for clinical relapse at 96 weeks after nucleos(t)ide analogues discontinuation.

|  | Sensitivity | Specificity | Positive predictive value | Negative predictive value |
| --- | --- | --- | --- | --- |
| SCALE-B |  |  |  |  |
| <260, N=14 | 96.8% | - | - | 92.9% |
| ≥320, N=37 | - | 73.8% | 56.8% | - |
| HBV RNA |  |  |  |  |
| < 2 log_10_  copies/mL, N=45 | 64.5% | - | - | 75.6% |
| > 2 log_10_  copies/mL, N=47 | - | 55.7% | 42.5% | - |
